# Differences in Power Spectral Densities and Phase Quantities Due to Processing of EEG Signals

**DOI:** 10.3390/s20216285

**Published:** 2020-11-04

**Authors:** Raquib-ul Alam, Haifeng Zhao, Andrew Goodwin, Omid Kavehei, Alistair McEwan

**Affiliations:** 1 School of Electrical and Information Engineering, University of Sydney, Sydney, NSW 2006, Australia; 2 School of Biomedical Engineering, University of Sydney, Sydney, NSW 2006, Australia; haifeng.zhao@sydney.edu.au (H.Z.); a.goodwin@sydney.edu.au (A.G.); omid.kavehei@sydney.edu.au (O.K.); alistair.mcewan@sydney.edu.au (A.M.); 3 The University of Sydney Nano Institute, The University of Sydney, Sydney, NSW 2006, Australia

**Keywords:** electroencephalography, spectral analysis, visual evoked potentials, pipelines, correlations

## Abstract

There has been a growing interest in computational electroencephalogram (EEG) signal processing in a diverse set of domains, such as cortical excitability analysis, event-related synchronization, or desynchronization analysis. In recent years, several inconsistencies were found across different EEG studies, which authors often attributed to methodological differences. However, the assessment of such discrepancies is deeply underexplored. It is currently unknown if methodological differences can fully explain emerging differences and the nature of these differences. This study aims to contrast widely used methodological approaches in EEG processing and compare their effects on the outcome variables. To this end, two publicly available datasets were collected, each having unique traits so as to validate the results in two different EEG territories. The first dataset included signals with event-related potentials (visual stimulation) from 45 subjects. The second dataset included resting state EEG signals from 16 subjects. Five EEG processing steps, involved in the computation of power and phase quantities of EEG frequency bands, were explored in this study: artifact removal choices (with and without artifact removal), EEG signal transformation choices (raw EEG channels, Hjorth transformed channels, and averaged channels across primary motor cortex), filtering algorithms (Butterworth filter and Blackman–Harris window), EEG time window choices (−750 ms to 0 ms and −250 ms to 0 ms), and power spectral density (PSD) estimation algorithms (Welch’s method, Fast Fourier Transform, and Burg’s method). Powers and phases estimated by carrying out variations of these five methods were analyzed statistically for all subjects. The results indicated that the choices in EEG transformation and time-window can strongly affect the PSD quantities in a variety of ways. Additionally, EEG transformation and filter choices can influence phase quantities significantly. These results raise the need for a consistent and standard EEG processing pipeline for computational EEG studies. Consistency of signal processing methods cannot only help produce comparable results and reproducible research, but also pave the way for federated machine learning methods, e.g., where model parameters rather than data are shared.

## 1. Introduction

Electroencephalography (EEG) signals have long been studied for various applications to understand brain dynamics from the underlying electrical activity of neurons. Among them, modern computational studies, including pre- or post-stimulus event-related potential (ERP) analysis [1,2,3,4,5,6], pattern recognition [7,8], cortical generator estimation by interpreting oscillations, is frequency bands [9,10], event-related synchronization/desynchronization [11], brain-computer interface [12], etc., are particularly popular among EEG researchers. In these applications, an array of signal processing methods have been applied to EEG signals over the past two decades many of which were summarized by Bashashati et al. [13]. Using the processing methods, an array of features is usually extracted from the EEG signals [14,15,16,17,18,19,20]. The features are then used for various tasks such as statistical analysis or machine learning based methods [14,15,16,17,18,19,20,21]. The EEG signal processing methods, in themselves, have a number of selection parameters, which have been found to be selected inconsistently across different studies. Examples can be found in a range of EEG research territories, including EEG classification studies that utilize autoregressive models or machine learning methods [14,22,23,24]. For example, in a visual evoked potential study, Faust et al. used and contrasted multiple auto-regressive models to estimate power spectral density (PSD) [22,25], while Sharmilakanna et al. used Welch’s method (non-parametric) in their study for the same task with the same dataset [23,26]. Welch’s method is also found in a variety of other EEG studies [27,28]. Moreover, Jaworska et al., Thatcher et al., and Schack et al. used the Fast Fourier Transformation (FFT) method for power estimation in their studies [14,24,29]. In other signal processing domains, such as studies dealing with analyzing the effect of pre-stimulus EEG on motor evoked potentials (MEP) caused by Transcranial Magnetic Stimulation, methodological differences for PSD estimation, filtering, etc., have been found. For example, İşcan et al. used Fast Fourier Transformation (FFT), which is non-parametric in nature, to estimate frequency band PSD in the pre-stimulus time-window starting from −4.5 s and −0.5 s (0 s indicates the time of stimulation) [19]. On the contrary, for a similar goal, Hussain et al. used a parametric autoregressive model approach (Burg’s method) on the time-window of 150 ms [18,25,30]. These two studies produced inconsistent results. More specifically, İşcan et al. did not find any correlation between MEP amplitude and beta frequency band power but Hussain et al. did find such correlations. Since different datasets were used in these studies, it is difficult to know the cause of these inconsistencies as they may just reflect sampling or biological variability. The inconsistencies may also be the product of methodological differences. To investigate this, it might be helpful to perform different processing methods on the same dataset.

In general, Welch’s method (a modified approach of FFT), FFT method, and Burg’s method may be regarded as the three most widely used methods for PSD estimation within a frequency band in EEG signal processing publications. These three methods, despite appearing to serve the same purpose, are mathematically different, resulting in slightly different outcomes as shown by Xiao-Yu et al. [31]. A consistent choice of one of these three methods may reduce inconsistencies across studies producing comparable results. Furthermore, the choice of time-windows can strongly influence the Fourier transformations [32]. Otherwise, the different interactions between these choices may play a role in producing differences in results across studies, similar to the differences, which were found between the two studies by İşcan et al. and Hussain et al., respectively [18,19]. Although authors often regard such methodological differences to be a potential cause for the observed differences in results, the alternative choices of the selection variables were deeply underexplored. A comparison of the effects of these selection variables remains to be examined. In a recent study, Robbins et al. compared the effects of different EEG processing pipelines on signal statistics and spectral characteristics [33]. In their study, it was evident that the spectral features, particularly in the low frequency range, are affected significantly depending on which pipeline was employed. However, the study was limited to differentiating the entire preprocessing pipelines ignoring finer variable steps within the pipelines, such as filter selection, PSD estimation method selection, time-window selection etc.

Furthermore, the choices available in the EEG transformation method may also prove consequential for PSD estimation. İşcan et al. used mean EEG signals averaged across all electrodes [19], while in a similar study, Hussain et al. utilized the Hjorth transformed signal of the C3 electrode for their analysis [18]. Average signals reflect the overall state of the brain, reducing spatial resolution, while Hjorth transformed signals indicate the scalp current density, which offers better spatial resolution with more independent quantities [34]. The results generated by the two studies were found to be inconsistent. Specifically, Hussain et al. found that the pre-stimulus beta frequency band influences the MEP amplitude, whereas İşcan et al. did not find such correlation. As these two studies have many methodological differences, including PSD estimation method selection, Hjorth transformation application, and time-window selection, it is difficult to know which of the methodological differences may have contributed to the inconsistencies in the results.

The choice of filters, required to eliminate 50 Hz or 60 Hz line noise and to extract sub-frequency bands, was different across studies. The two most popular choices for the filters are Butterworth filter (Infinite Impulse Response [IIR] filter) [35,36,37,38] and Blackman–Harris window filter (Finite Impulse Response or FIR filter) [18,39,40]. Being fundamentally different [41], the choices of these two filters may affect the PSD quantities. A contrast of filters and their interaction with other selection variables (e.g., time window, PSD estimation method) may also prove useful in explaining differences in results in cross-studies.

In many studies artifact removal pipelines were used to eliminate artifactual components automatically from the EEG traces [42,43]. However, in other studies, manual rejection of artifactual trials was carried out [18]. This difference may be the source of further inconsistencies.

Similar to PSD quantities, phases are also considered as an important indicator in computational EEG studies since phases reflect the state of the brain and phase patterns can reflect selectivity of neural firing [38,44]. The oscillatory phases of different frequency bands can be estimated in a number of ways [38,45]. Usually, in phase analysis experiments, the phase values are estimated using one of three methods: Complex Demodulation [46], Hilbert transformation [47], and In-Phase/In-Quadrature Filtering [45]. Similar to the selection of the PSD estimation method or time-window, the selection of these phase estimation methods may also affect the outcome in studies dealing with phases. However, these three methods for phase estimation have already been contrasted by Ktonas et al., and, hence, are beyond the scope of the current study [45]. Nonetheless, it may be hypothesized that choices in other preprocessing steps, such as artifact removal, EEG transformation, and filters, may influence phase quantities.

Methodological differences not only make it difficult to compare results, they also pose a problem assessing reproducibility of machine learning models across restricted datasets. Sharing sets of model parameters may be easier if consistent data processing methods are used across studies. The study hypothesizes that the selection of the above-mentioned methods/factors, specifically artifact removal options, EEG transformation options, filter choices, time window choices, PSD estimation method choices, and their interactions may influence the output power and phase quantities. If so, some assumptions made in past studies, which accounted methodological differences for inconsistencies in the results, might be validated. Therefore, two options of artifact removal, three choices of EEG transformation methods, two options of filters, two choices for time-windows, and three possible choices for PSD estimation methods were tested against the output measures: powers and phase quantities. The interaction effects of the said selections were also tested. To validate our results, the analyses were run on two different EEG datasets, which were recorded in two different experimental settings. The EEG recordings in the first dataset included signals from subjects in a visual stimulation experiment. The second dataset contained resting-state EEG signals. Finally, statistical analyses were performed separately for each dataset and the effects of the selections were contrasted.

## 2. Materials and Methods

### 2.1. Participants

The first dataset, namely Dataset 1, was obtained from University of California, Irvine Knowledge Discovery in Databases (UCI KDD) archive. The dataset included EEG recordings from 45 healthy control participants and 77 alcoholic participants [48]. For the purpose of the current study, recordings only from the healthy control subjects were analyzed. The number of male (24.3 ± 3.1 year old) and female (23.2 ± 1.7 year old) participants were not made public. All subjects in Dataset 1 were right-handed and without any known neurological history. A more detailed description of the demography can be found at the dataset repository [48] and the associated study [49]. Dataset 2, also available publicly, was collected by Trujillo et al. Dataset 2 included EEG recordings from sixteen Texas State University undergraduate students (8 female, 8 male, mean age = 21.8 ± 8 years, age range = 18 to 26) [50,51].

### 2.2. Experimental Settings: Dataset 1

EEG recordings were obtained from the participants by placing 64 electrodes at standard sites [48] of 10/20 International montage (Standard Electrode Position Nomenclature, American Electroencephalographic Association 1990). Zhang et al. described in detail the data collection process for this dataset in their study [49]. The participants were relaxed in an awake state with eyes open. All scalp electrodes were referenced to Cz. Subjects were grounded with a nose electrode, and the electrode impedance was always kept below 5 kOhms. The signals were amplified with a gain of 10,000 by Ep-A2 amplifiers (Sensorium, Inc., Charlotte, VT, USA) with a band-pass filter (0.02 Hz and 50 Hz) and recorded on a Concurrent 55/50 computer. The amplified EEG signals were recorded with a sampling frequency of 256 Hz. This dataset was originally collected by Zhang et al. primarily to examine how EEG correlates to alcoholism. The participants were subjected to visual stimulations 40–50 times after which they performed a categorization task. In total, the dataset contained 120 trials including three conditions. The first condition was the visual stimulation and the latter two were recorded when the participants performed motor operation as a response to the stimulus. Only the trials of non-alcoholic subjects were used in this study to remain consistent with the other dataset.

### 2.3. Experimental Settings: Dataset 2

The experimental setup of Dataset 2 was described in greater detail in the associated study [50]. A 72 channel EEG cap with an extended 10/20 International montage was used to record EEG signals from each participant during resting state and categorization task (BioSemi Active II system; 24-bit DC mode; 2048 Hz initial sampling rate down-sampled online to 256 Hz; common mode sense reference electrode located between sites PO3 and POZ). No ground location, impedance, amplifier configuration, and mode of the recording (alternating current [AC] or direct current [DC]) was reported in the associated study for Dataset 2. Nevertheless, since the study dealt with relative changes occurred from selecting different choices, the mode of EEG recording was not relevant. For the purpose of current study, only the resting state EEG was used which included 8.5 min of EEG recording. Dataset 2 with resting state EEG is fundamentally different from Dataset 1 with visual stimulation, providing a means to validate the results obtained from Dataset 1 in a different domain of EEG.

### 2.4. Data Analysis

#### 2.4.1. EEG Preprocessing

**Figure 1:**
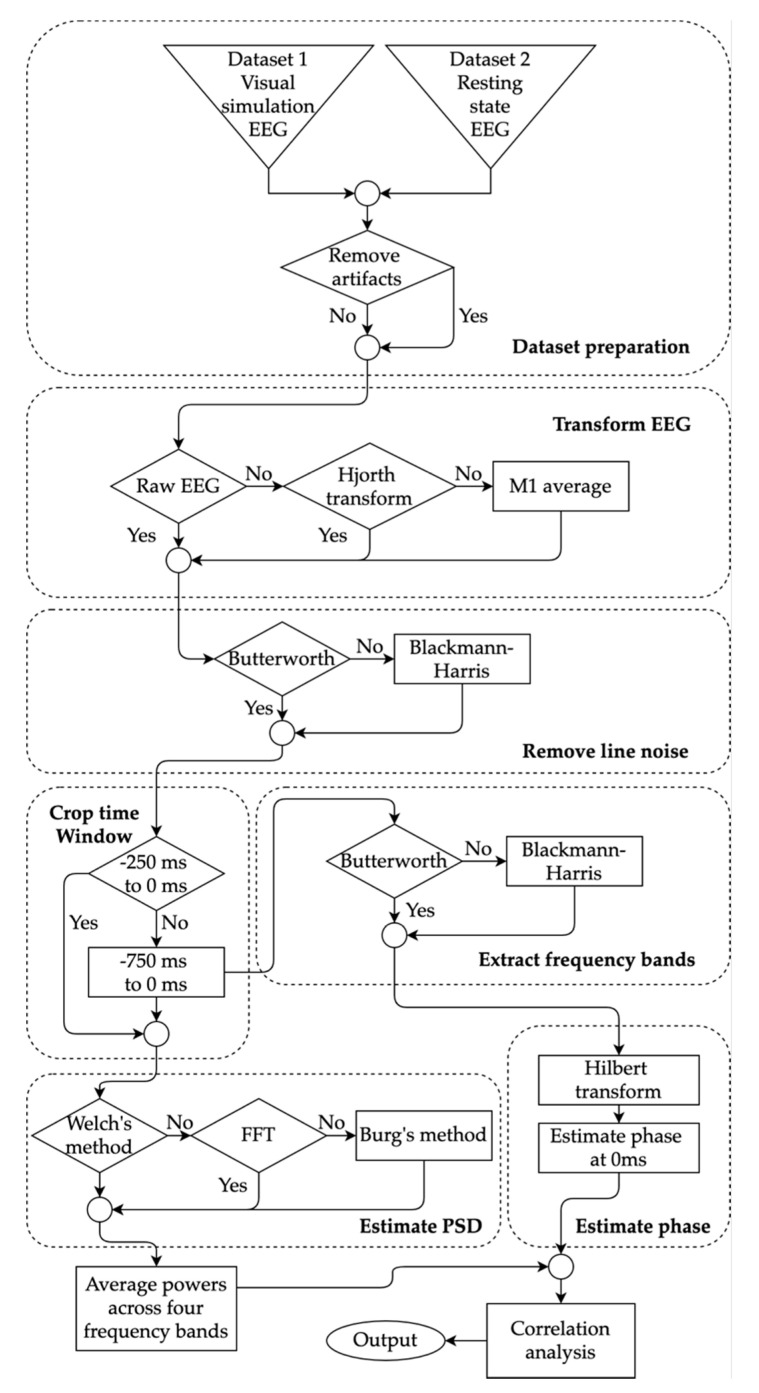
Flow diagram of the processing methods carried out to estimate powers and phases for four frequency bands. The selection choices of fives methods were explored highlighted with diamond shape: artifact removal, electroencephalogram (EEG) transformation, filtering, time window selection, and power spectral density (PSD) estimation. The estimated powers and phases were used to find the correlation between the choices.

All of the processing methods for both datasets are summarized in Figure 1. Collected EEG recordings were preprocessed in MATLAB 2019b (MathWorks, Natick, MA, USA) using the open-source EEGLAB toolbox version 2019.0 (sccn.ucsc.edu/eeglab) [52]. EEG signals in Dataset 1 were already epoched in the source repository where each EEG trial was kept in a separate file that contained 1 s of the signal. Therefore, no additional epoching was required for Dataset 1. In Dataset 2, the resting state EEG signals were epoched with 1 s intervals which resulted in ~510 trials for each participant. For each subject in both datasets, all of the trials were loaded in EEGLAB and correct electrode location was then assigned for all channels. All signals from both datasets were band-pass filtered within 2 Hz and 80 Hz using Hamming windowed sinc FIR filter (automatic filter order using EEGLAB’s pop_eegfiltnew function). The lower cutoff of 2 Hz was chosen based on Dimigen’s suggestion to later have proper component decomposition [53]. The upper cutoff was chosen so that the gamma frequency band can be used in the analysis.

To assess the effect of artifact removal, two choices were explored in both datasets: with and without artifact removal. For the first choice, an EEGLAB based automatic artifact rejection pipeline ADJUST, developed by Mognon et al. was used [54]. The first step in this method was to run independent component analysis (ICA) and extract different components of the signals [39,55]. To properly decompose the components, Onton et al. suggested that the number of extracted components, n should be such that $n^{2}k=$ number of time points, k≫20 [56]. The number of components, n, is equal to the number of channels (64 for Dataset 1 and 72 for Dataset 2) in a square matrix paradigm (Infomax). Since the length of the signals in Dataset 1 was only ~120 s, the number of time points were much less than the required amount. A solution was to reduce the number of components along with the number of channels. Therefore, the number of channels was reduced from 64 to 32. To this end, 10/20 international montage 32 channels were selected from all the channels in both datasets. In this way, the number of data points were sufficient for extracting 32 components in both datasets. Subsequently, ICA with Infomax algorithm [57] was carried out to extract the components from the concatenated trials. The ADJUST pipeline then computed a series of spectral and temporal features from the extracted components. The ADJUST package included a classifier based on the expectation maximization technique, which was used to classify and reject the components related to eye blinks, vertical, and horizontal eye movements and general discontinuities [58]. After rejection, the EEG signals were reconstructed with the remaining components. Further details about the ADJUST algorithm are described in the associated study [54]. In Dataset 1, trials of second and third conditions (discussed above) were rejected. The remaining trials did not contain any motor evoked potentials and were used for analysis. This allowed for having consistent trial types (no motor evoked potentials in motor cortex) from both datasets for the analysis. Each trial on both datasets was one second long which was labeled −1 s to 0 s in the time axis. Here, 0 s represents the end of the trial. This labeling convention was adopted to remain consistent with any pre-stimulus signal analysis studies where 0 s usually represents the time of stimulation.

#### 2.4.2. EEG Transformation

The EEG signals from both datasets were transformed to obtain two additional types of signals. This allowed us to compare the effect of these three EEG signal types on powers and phases. In the first transformation, the raw signals were transformed into current source densities (CSD) (in μV/cm), using the Hjorth transformation implementation [59] available in the FieldTrip toolbox for MATLAB [60]. For each subject, the signals were Hjorth transformed using C3 as the central channel and FCz, CPz, FT7, and TP7 as the neighboring channels. C3 channel, being a part of motor cortex, is the primary channel for most motor evoked potential analysis studies. The trials selected for this study (Dataset 1 and Dataset 2) did not include any motor tasks. Therefore, focusing on the motor cortex allowed for reducing variability across the datasets. In the second transformation, the EEG signals were averaged with a focus on the left motor cortex region. The average signal was calculated by averaging the channels FT7, FC3, FCZ, T7, C3, CZ, TP7, CP3, and CPZ. The two transformations resulted in a total of three types of signals: C3 raw signals (not transformed), C3 Hjorth transformed signals, and left motor cortex averaged signals. All signals were then passed through a notch filter to remove line noise between frequency band 48Hz to 52Hz for Dataset 1 and 58Hz to 62Hz for Dataset 2 [61,62]. For this purpose, one of two choices of notch filters were used interchangeably to compare their effects on the power levels. The first filter was an IIR Butterworth stop band filter of order 3. A FIR filter, Blackman–Harris (order 51 or 101 depending on the cropped time-window of EEG) with delay correction was selected as the second filter [63].

#### 2.4.3. Frequency Band Power Estimation

The three types of notch-filtered waveforms: raw EEG signals, Hjorth transformed signals and averaged signals were further processed in the Python programming language (version 3.7.3). The Power spectrum (in dB) was estimated from the filtered signals within two different time windows: −250 ms to 0 ms and −750 ms to 0 ms. The shorter time window of length 250 ms was chosen to cover at least a full cycle of oscillatory activity over 4 Hz (theta frequency band) and to reflect small time windows used in many studies [18]. The longer time window of 750 ms was selected to reflect longer time windows in previous studies [19]. The full 1 s windows were not used because rejecting the remaining segment of the 1 s trial allowed for eliminating continuity in the cropped trials potentially reducing causal effects across the trials. The order of the Blackman–Harris filter applied in the previous section depended on the time-window. In the case of −250 ms to 0 ms time-window, the order was 51. For the latter time-window, the filter order was 101. The power estimation was then carried out using three separate methods. Firstly, Burg’s method of order 64 and final data length of 256 [64] was used to estimate the power spectral density. Secondly, fast Fourier transform (FFT) method with normalization (length of Fourier transform = 128) was utilized to estimate the powers [65]. Lastly, the Welch’s method was used for power estimation [26]. The resulting spectrum was converted to decibel quantities and averaged across the range of each of the four frequency bands: theta (4 Hz to 8 Hz), mu (8 Hz to 12 Hz), beta (13 Hz to 30 Hz), and gamma (30 Hz to 80 Hz). The delta band was not included in the analysis since it would require either a time-window longer than 250 ms or signals of higher sampling frequency (Rayleigh frequency) for proper analysis. This produced four PSD quantities (theta, mu, beta, and gamma) for each trial and for each path of methods shown in Figure 1.

#### 2.4.4. Frequency Band Phase Estimation

Raw, Hjorth transformed and motor cortex averaged signals were band-pass filtered separately to obtain signals in the theta, mu, beta, and gamma bands [63]. To this end, the same two filters were used again, which were used for line noise removal. However, this time, the filters were band-pass filters instead of a notch filter. Similar to the methods employed by Hussain et al. and Wolff et al., the filtered signals of the four frequency bands were Hilbert’s transformed to obtain phases (in degrees) from −750 ms to 0 ms [18,47,55]. The shorter time window of length 250 ms was not selected during phase estimation because a signal of length 250 ms will contain only 0.6 to 1.2 cycles of the theta frequency band potentially resulting in inaccurate phases. The phase at 0 ms was selected (in degrees) for statistical analysis.

### 2.5. Statistics

For both datasets, the selection effects of five methods (factors) were assessed: artifact removal, EEG transformation, filters, time windows, and PSD estimation methods. Their effects on the resulting powers and phases were visualized using several plots. To analyze group level effects, Pearson correlation coefficient of PSD was computed for each pair of choices within each method [66]. This operation was performed for all subjects in both datasets, taking $2n_{i}$ quantities per subject where n is the number of trials in subject i, and multiplication by 2 indicates a pair of choices. Since three factors (artifact removal, filter, and time-window) out of five had two choices and two factors (EEG transformation, PSD estimation method) had three choices, taking any 2 choices within each method resulted in 3×C 22+2×C 32=9 sets of correlation coefficients for each dataset. All Pearson correlation coefficients were visualized in box plots to understand group level effects. These coefficients only showed the variation of the values. Therefore, to also understand whether the choices produced significantly different PSDs, the linear mixed effects model analysis was carried out. Separate models were analyzed for the four different frequency bands. Five factors and four frequency bands resulted in a total of 20 models for each dataset. Each model assessed one factor while the remaining four factors were kept constant. The constant values, which roughly represent the most widely used values, were as follows: artifact removal = yes, EEG transformation = raw, filter = Butterworth, time window = 750 ms, and PSD estimation method = Welch’s method. In the analysis of these models, data from all subjects were included selecting the power as the response variable, method choice as the fixed factor and subject number as the random effect variable (power ~ factor + [1|subject]). Finally, repeated measures ANOVA was performed on the models and all of the model assumptions were tested for conformity.

Pearson correlation coefficients were also computed for phase quantities. However, estimated phases showed a bimodal distribution with two peaks at around 90° and 270°, respectively. Therefore, they were divided into two groups to have the phases conform to a normal distribution within the groups. The two groups were named “peaks” (phase < 180°) and “troughs” (phase ≥ 180°). The correlations were measured separately for peaks and troughs. Since phase estimation pipeline had three factors (artifact removal: two choices, EEG transformation: three choices, and filter: two choices), this resulted in 2(2×C 22+1×C 32)=10 sets of correlation coefficients for each dataset. Linear mixed effect model analysis was carried out separately for each phase group, factor, and frequency band which resulted in a total of 24 models for each dataset. All subjects were included in the analysis where phase was selected as the response variable, method choice was chosen as the fixed effect variable and the subject number was selected as the random effect variable (phase ~ factor + [1|subject]). Subsequently, repeated measures ANOVA was performed on each of the models. Finally, the linear mixed effects model assumptions were tested for conformity.

## 3. Results

In Dataset 1, 45 healthy volunteers participated who performed object recognition tasks. From each participant, 40–50 trials were used for this study depending on the availability of the trials in the dataset. EEG of some trials included errors which were pre-specified in the description of the dataset repository [48]. The error prone trials were rejected for analysis. 16 healthy volunteers participated for Dataset 2. Files of one participant had reading errors which were rejected for further analysis.

PSDs and phases were estimated for all trials in both datasets. Effect of the choices available in five methods on PSDs were assessed: artifact removal, EEG transformation, filters, time windows, and PSD estimation methods. Similarly, the choices in three methods were assessed to see how they modulated phases: artifact removal, EEG transformation types, and filters.

### 3.1. Variation of Power Spectrum Density

**Figure 2:**
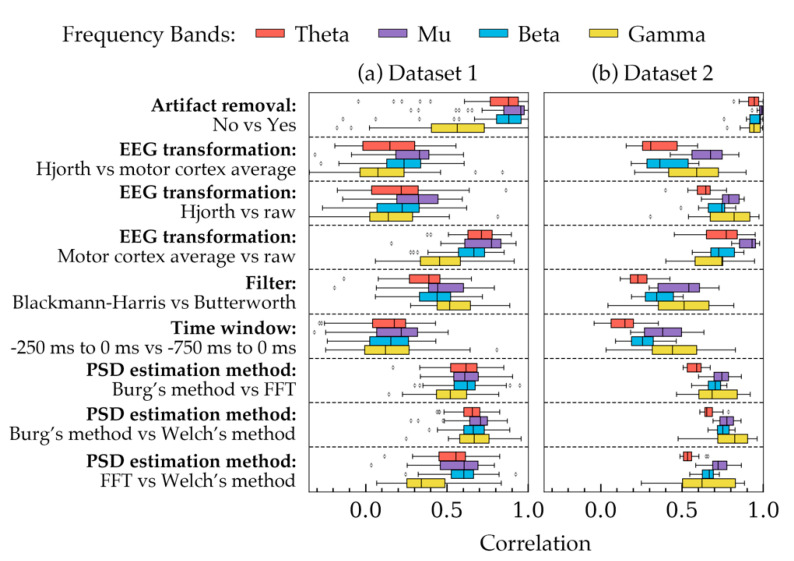
Correlations between different choices of each method are demonstrated for all subjects in both datasets. Each frequency band was considered separately while calculating the correlations. The four bands are highlighted with four different colors. For each method (presented in each row), the remaining methodological choices were kept constant as follows: artifact removal = yes, EEG transformation = raw, filter = Butterworth, time window = 750 ms, and PSD estimation method = Welch’s method. The boxes indicate the quartiles of the samples while the whiskers extend to show the rest of the distribution up to 1.5 inter-quartile range. The dots indicate the outliers. (**a**) The correlations obtained from all subjects of Dataset 1. (**b**) Correlations obtained from all subjects in Dataset 2.

Figure 2 shows the correlation coefficients calculated for all subjects in both datasets. Each row, separated by dashed lines, shows how different choices available in a method were correlated with each other across the four frequency bands. In each row, the choices of the remaining four methods were kept constant. The constant choices roughly represent the most widely used choices in EEG processing studies: artifact removal = yes, EEG transformation = raw, filter = Butterworth, time-window = 750 ms and PSD estimation method = Welch’s method. From Figure 2, four important aspects can be observed. Firstly, the coefficients had an overall similar trend in both datasets despite some small differences, particularly in the PSD of gamma frequency band. Secondly, PSDs in two rows seemed to be highly correlated: with vs. without artifact removal, and average vs. raw signal. The remaining choices influenced the PSDs in greater strength as they had smaller correlations in the figure. Thirdly, the correlations in the gamma frequency band varied significantly in both datasets as they had the most standard deviation. Lastly, the choices, in general, seemed to affect the PSDs more as the frequency was decreased (except gamma band). As a result, smaller correlations were found in the theta band in most cases.

Linear mixed effect model analysis was carried out on data from all subjects to determine how significant the variations in PSDs were. The result of the analysis shown in Table 1. The table indicated that most choices had significant effects (*p* < 0.05) on the PSD values obtained from both datasets. However, filter choice did not show significant effect (*p* > 0.05) on the powers of theta and mu frequency bands in Dataset 1. Additionally, the choice of time window size did not seem to affect theta and mu band powers in Dataset 1. Choices in artifact removal did not result in significantly different PSDs of mu band in Dataset 2.

Table 1 showed that most choices in the five methods had significant effects on PSD depending on the frequency bands. On the contrary, Figure 2 indicated that EEG transformation and time-window had the least correlated powers. Based on these two results, it may be suggested that EEG transformation and time-window have the most effect on PSD quantities. The choices of the remaining methods, despite showing significant mean change (*p* < 0.05) in the power values, were somewhat correlated (Figure 2). Therefore, in a trial-to-trial study, their effects may not be considered as pronounced as the choices in EEG transformation and time-window.

**Figure 3:**
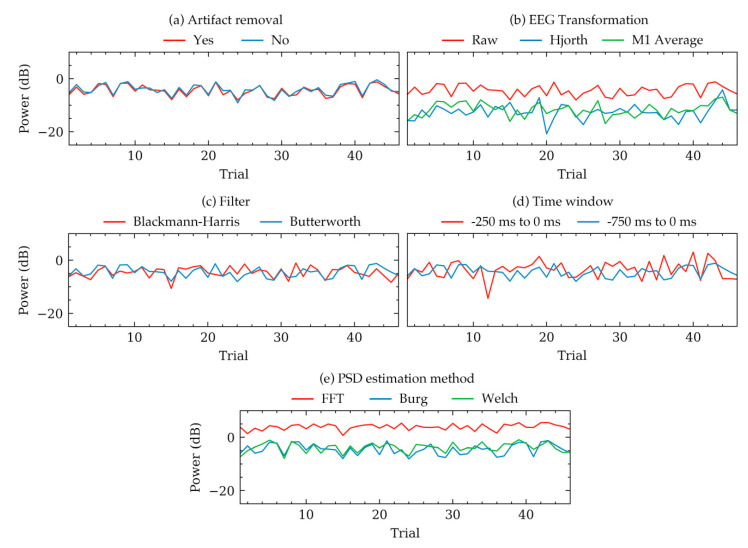
Example variations of PSD quantities of beta band calculated from 46 trials of a random subject in Dataset 1. Each subplot shows how the PSD varied for this subject depending on the selected choices within five methods. Each subfigure contrasts choices in one method keeping the other methods constant to default values. The default constant values were as follows: artifact removal = yes, EEG transformation = raw, filter = Butterworth, time window = 750 ms, method = Welch’s method. (**a**) Variations for artifact removal choices. (**b**) Variations for EEG transformation choices. (**c**) Variations for filter algorithm choices. (**d**) Variations for time window choices. (**e**) Variations for PSD estimation method choices.

**Figure 4:**
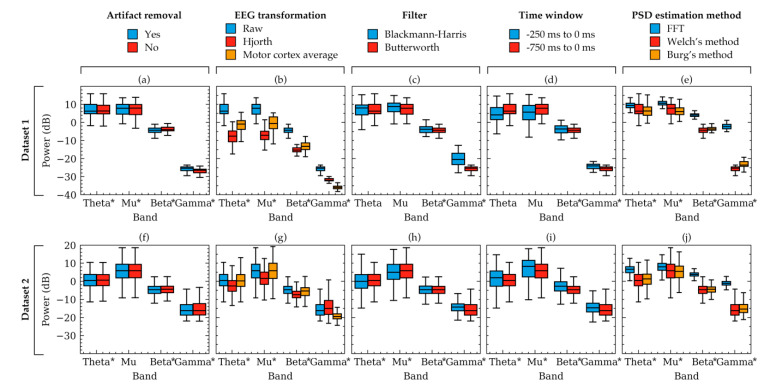
Box plots showing example power variation within two subjects. The variations occurred due to the selection of choices available in artifact removal (**a**,**f**), EEG transformation (**b**,**g**), filter (**c**,**h**), time-window (**d**,**i**), and PSD estimation method (**e**,**j**). Powers were computed from the EEG signals of two random subjects from Dataset 1 (**a**–**e**) and Dataset 2 (**f**–**j**), respectively. Each subfigure represents the effect of choices available in a factor for the subject. The other factors were kept constant as follows: artifact removal = yes, EEG transformation = raw, filter = Butterworth, time window = 750 ms, method = Welch’s method. The boxes indicate the quartiles of the samples while the whiskers extend to show the rest of the distribution up to 1.5 inter-quartile range. Significant models from Table 1 are indicated in the figure using asterisks (*) near the frequency band names.

Figure 2 and Table 1 demonstrates group level variations of PSDs. To illustrate some examples of these variations within individual subjects, Figure 3 and Figure 4 were plotted. Figure 3 shows the trial to trial power variations within the beta frequency band of a random subject in Dataset 1. The variations were the result of selecting different choices within each of the five factors of signal processing methods. Each subfigure in Figure 3 was plotted to contrast choices in a method while all other method choices were kept constant. The constant values of the choices were the same as that in Figure 2. Figure 3 indicated that there were variations in some trials, even though the overall trend of the powers were same for most choices. Choices in artifact removal and filters seemed to produce least variations in trials. EEG transformation and PSD estimation method choices seemed to produce an offset, particularly when Hjorth transformation (Figure 3b) and FFT method (Figure 3d) were used. Time window seemed to produce drastically different PSDs (Figure 3d). Visualization in Figure 3 was mostly consistent with the results obtained from Figure 2 and Table 1, which showed that the choices in EEG transformation and time-window produced the most pronounced variations (ignoring offsets) of power. However, unlike Figure 3, the models in Table 1 indicated significant variations can arise due to the choices in artifact removal.

Figure 3 showed the powers of beta band only. To visualize some examples of the other frequency bands, power boxplots of each frequency band of a subject from each dataset were plotted in Figure 4. Constant values were again chosen to plot each box and the rationale for the constant values is the same as that for Figure 2. These boxplots also verified the results obtained from Table 1 and Figure 2 which suggested that variation in the choice of EEG transformation and time-window produces the strongest variation (ignoring offsets) in power.

### 3.2. Variation of Pre-Stimulus Phase

**Figure 5:**
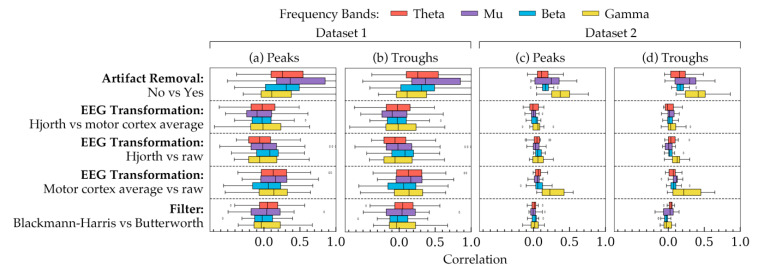
The correlation coefficients, calculated using phases from all subjects, showing how choices in each method are correlated to each other. The coefficients are shown for (**a**) the peak phase trials within all subjects in Dataset 1, (**b**) the trough phase trials within all subjects in Dataset 1, (**c**) the peak phase trials within all subjects in Dataset 2, and (**d**) the trough phase trials within all subjects in Dataset 2. Each row, separated by a dashed line, represents the correlation between a pair of choices within a method. The remaining two methods were kept constant for each row. The constant values were as follows: artifact removal = yes, EEG transformation = raw, filter = Butterworth. The four frequency bands are highlighted in four different colors. The boxes indicate the quartiles of the samples while the whiskers extend to show the rest of the distribution up to 1.5 inter-quartile range. The dots indicate the outliers in the figure.

Figure 5 shows the correlation coefficients between all possible choices in the methods of phase estimation. The two phase groups are shown in different subplots: peaks (Figure 5a,c), troughs (Figure 5b,d). Taking all subjects into account, each row shows how the effects of choices in a method were correlated with each other. The remaining methods were kept at a constant choice the rationale for which is the same as that of Figure 2. In Figure 5, a difference in standard deviation was evident between the two datasets: phases of Dataset 2 showed smaller standard deviations than that of Dataset 1. However, overall, both of the datasets showed similar trends in the correlation plots with an exception in the gamma frequency band. Additionally, Figure 5 shows that artifact removal, in this case, had the least effect on phase estimation, since with and without artifact removal seemed to result in relatively stronger correlations between the phases.

To understand how significant the effects of the three factors on the phase quantities are, 24 linear mixed effect models were analyzed with data from all subjects of both datasets. The results of the models are shown in Table 2. The table indicated that the choice of filters had the most significant effect (*p* < 0.05) on phase values. Artifact removal choices did not have significant on the phases (*p* > 0.05). The effects of choices in EEG transformation seemed to vary between the two datasets, particularly in theta and gamma bands. Overall, the results from Figure 5 and Table 2 indicated that filters and EEG transformation can affect the phase quantities, the nature of which depends on the frequency band.

**Figure 6:**
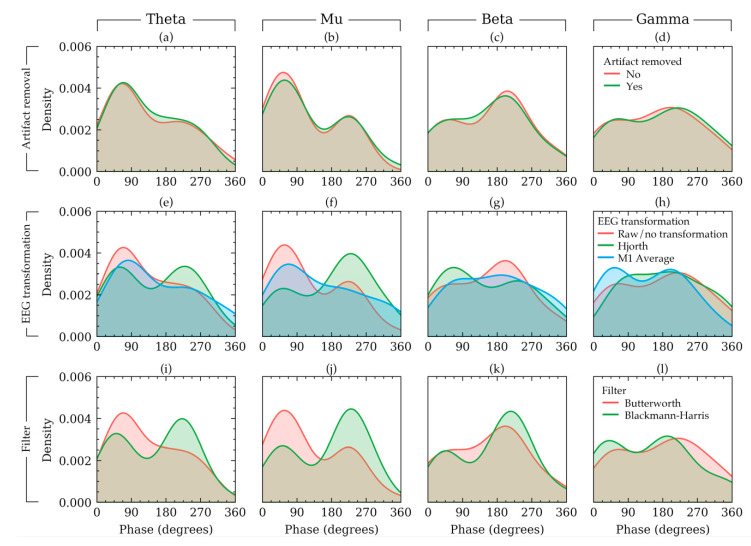
The density plot of phase values of a random subject in Dataset 1 illustrating how the choice of artifact removal (**a**–**d**), EEG transformation (**e**–**h**), and filter (**i**–**l**) affected the phase distribution for that subject. The distributions are plotted separately for four frequency bands in each column. The effect of each factor is plotted in each row keeping the other remaining factors constant with constant values. The constant values were as follows: artifact removal = yes, EEG transformation = raw, filter = Butterworth filter.

To visualize some examples effects of the three methods on the phases of an example subject, the density plot of the phases of EEG signals of a random subject in Dataset 1 is presented in Figure 6. In this figure, each row shows the effects of choices available in a method keeping the other methods constant. The rationale for the constants is the same as that of Figure 2. Figure 6 shows that the phases had a bimodal distribution. It also shows that the choices in EEG transformation and filter affected the output phase values nonlinearly across the four frequency bands. For example, in beta frequency band (Figure 6g), if raw signals were used, the trough phase trials outnumbered the peak phase trials. However, such phenomenon was not apparent for the other frequency bands (Figure 6a,b,d). Similar non-linearity can be found for filter selection (Figure 6i–l). The visualization of Figure 6 agrees with the results from Table 2 and Figure 5, which indicated that EEG transformation and filters affect the phase quantities substantially. The choices in EEG transformation and filter algorithms produced substantially different distributions for the subject in Figure 5. 

## 4. Discussion

The goal of the current study was to contrast the effects of different methodological choices that arise in a typical EEG processing study dealing with EEG power and phase quantities. The selection of these choices varied across past studies potentially introducing disproportional results. The study hypothesis, whereby methodological differences can account for some of the inconsistencies in EEG studies, was not rejected as some choices seemed to affect the power and phase values across frequency bands. More specifically, the results indicated that the choices within EEG transformation method and time window can influence PSD quantities most significantly. Artifact removal, filter, and PSD estimation method choices had relatively smaller effect on the PSDs. Similarly, EEG transformation and filter choices had significant effects on phase values. In some cases, the choices introduced offsets in the values which can be ignored in a study dealing with relative variations of power and phase. In some other cases, the results showed nonlinear variations across the frequency bands, indicating possible disagreements between studies with methodological differences. In short, the results signify that choices in EEG transformation, time window and filter in an EEG processing study can, more or less, influence the PSDs and phases.

### 4.1. Effects on Power Spectral Density

Artifact removal had small effects on the PSD values (high correlation in Figure 2). However, the effect was significant evident from model 1 to 4 from Table 1 (*p* < 0.05). The significance, which depends largely on the number of samples in the data, indicated that there was a relative mean power change and the correlations indicated that despite having different means (or offsets), the powers were correlated. In other words, the effect size was small but statistically significant. Visualization of the power of a random subject in Figure 4 showed that the beta and gamma band were affected slightly more than the other bands. This is likely because using the ADJUST pipeline, muscle artifacts and line noises were removed, which were reported to have morphologies in the beta and gamma frequency range [67].

EEG transformation had significant effects (Table 1) on the PSDs. Accordingly, Figure 2 showed relatively small correlation of powers between the three choices in EEG transformation. Therefore, it may be suggested to have a standardized choice made across EEG studies of similar context to avoid inconsistencies in results.

Filter selection produced significant variation of PSDs for particular frequency bands: beta and gamma band of Dataset 1 and all bands of Dataset 2. This difference between the dataset is most likely because the signals in Dataset 1 were already band pass filtered (0.2 Hz to 50 Hz) in the source repository. However, Dataset 2 did not include pre-filtered signals. Additionally, after artifact removal, Dataset 1 included signals from 2 Hz to 50 Hz, whereas signals in Dataset 2 included data up to 80 Hz. Therefore, the effect of the filters seemed to depend on the dataset and a conclusion could not be made as to whether the choice of filters can significantly affect PSDs.

The time window selection affected PSDs significantly except in the mu and beta band of Dataset 1 (Table 1, model 13 to 16). This difference between the datasets may have occurred because subjects in Dataset 1 performed motor tasks in the rejected trials where the subjects clicked mouse button to indicate which object they recognized. The trials used in this study did not include motor actions but they may have been involved in motor preparation, which was known to be the result of mu and beta rhythm desynchronization [68]. Time window choice seemed to result in relatively less correlated PSD values (Figure 2). This could be one of the reasons why Hussain et al. found significant correlations between beta powers and MEP amplitude while using small time windowed Hjorth transformed signals [18], but İşcan et al. did not find such correlation using longer average signals [19]. Therefore, it may be suggested to have a standardized time-window to remain consistent across studies.

Choices in PSD estimation method influenced the PSDs within all frequency bands (Table 1, model 17 to 20). However, Figure 2 indicated that although mean powers were significantly different (Table 1), the correlation between the powers were relatively stronger. Significant *p*-values and high correlation could mean that there were offsets in PSD values for individual PSD estimation method choices, but despite the offsets, the values were correlated. Some examples of the offsets are visualized in Figure 3e and Figure 4e,j. In a study dealing with the relative differences between the band powers or trials, these offsets may not contribute to a significant difference in the results as the relative variations across the frequency band are similar.

The correlations in the gamma frequency bands had larger standard deviation, unlike the other three bands (Figure 2). These correlations also differed substantially between the two datasets. This is partly because the source repository of Dataset 1 included filtered signals within 0.2 Hz to 50 Hz. However, for Dataset 2, the entire spectrum (2 Hz to 80 Hz) was available. As a result, the definition of the gamma frequency band was different for the two datasets [69]. Another reason might be that Dataset 2 had orders of magnitude larger gamma band line noise than that in Dataset 1.

The correlation plots also showed that the lower frequency bands, in general, have weaker correlations than the other bands (ignoring gamma band). This is consistent with what Robbins et al. reported in their study [33]. They suggested that more differences arise in the lower frequency bands because the signals may be affected by the downstream analysis of the lower frequency bands, which includes artifacts, such as large amplitude artifacts and eye blink artifacts. Additionally, since a low frequency component of a signal has a longer time-period, the signal contains fewer components of low frequency bands in a given time-window. Having fewer samples may cause the PSD estimation algorithms to render less accurate PSDs, resulting in weaker correlations. Therefore, care should be taken selecting parameters while analyzing lower frequency bands.

On the whole, the effects on PSDs suggest that researchers should be careful while making choices in EEG transformation and time-window since they seemed to have the most effects on PSDs. Artifact removal, filter, and PSD estimation method choices may have less effect on PSDs, which can possibly be ignored in trial-to-trial studies.

### 4.2. Effects on Phase

To see how choices in artifact removal, EEG transformation and filters can affect phase quantities, correlation analysis and linear mixed effect model analysis were carried out. The results indicated that choices in artifact removal had almost no effect on the phases (Table 2, model 1 to 4). Accordingly, Figure 5 showed relatively strong correlations between the powers resulted from the two choices in artifact removal. Visualization in Figure 6a–d showed subtle and insignificant changes in the phase distribution. Therefore, it may be concluded that artifact removal may not affect phase quantities.

Choices in EEG transformation produced weakly correlated phases (Figure 5). However, Table 2 indicated that these variations were significant only in particular cases. The variation occurred significantly in the phases of theta band (Dataset 1, model 5 and 17) and gamma band (Dataset 2, model 8 and both datasets, model 20). It is currently unclear why the variation occurs differently across datasets and frequency bands. Perhaps it is due to less accurate phase estimation in theta band (discussed above) and the differences in the gamma frequency band between the two datasets (also discussed above).

Although filter choices had small effect on the PSDs, they affected the phase quantities substantially (Table 2, model 9–12 and 21–24) except phases in gamma band of Dataset 1. Figure 5 is also in agreement with this since phases resulted in from different filters did not have strong correlations (mean correlation coefficient was approximately 0). It is difficult to know exactly what contributed to such significant differences. However, they were possibly because of the mathematical differences of how different filters operate as filters often have large and discontinuous gradients in the phase response even when their magnitude response is smooth. To summarize, the results showed that the choices in EEG transformation and filters altered the phase distribution of both of the datasets and hence, they should be carefully selected in such studies.

In short, the core finding of the current study was that the choices made for EEG transformation and time-window methods may influence the PSD quantities. Furthermore, EEG transformation and filters may affect the phase values of different frequency bands. Therefore, this study urges researchers to make consistent choices for these methods.

### 4.3. Challenges, Limitations, and Future Work

Some challenges and limitations must be acknowledged for the current study. This study was designed to assess the methodological differences in two different types of EEG experimental settings, one with visual stimulation and the other with resting state EEG. Although most of the results matched in both of the settings, some differences were evident, especially in the gamma frequency band. A limitation of this study was that more experimental settings were not tested and the differences across experimental settings were not validated in other datasets. To validate the differences, the same methods may be conducted on additional data similar to Dataset 1 or 2.

Although two different types of EEG datasets were used in this study, it may be advantageous to assess the method choices in other domains of EEG, such as EEGs with event-related potentials other than visual evoked potentials. The study also did not consider many other options available in the preprocessing methods due to brevity. In a future work, the remaining options may be explored such as the multitaper spectral estimation method [35] and the individual alpha frequency anchoring technique for power estimation [70]. Moreover, several popular preprocessing steps were avoided in the current study. For example, resampling seemed to be a common step in modern EEG studies with high sampling frequency datasets. However, in the current study, both of the datasets were recorded in 256Hz sampling frequency. This restricted the down-sampling of the signals since the sampling rate was needed to remain above Nyquist frequency [71]. It may be worthwhile to assess resampling and other commonly used methods. This study also did not assess the order of operations and how it impacts powers or phases. The order of operations may be an important factor influencing the results.

EEG signals in this research had low spatial resolution (32 channels). As a result, it was possible that potentials from brain regions other than motor cortex may have leaked into the Hjorth transformed channels and averaged motor cortex channels. A higher density channel montage could focus only in the primary motor cortex and, hence, reduce potentials from other brain regions that may vary between the datasets.

In this work, it was not attempted to find any mathematical relationship between the PSDs and phases resulted from distinct choices. A future study may investigate such relationships as function of the selected choices, which may result in a very useful transformation tool that could assist comparing transformed results across studies with methodological differences.

Although this study was not greatly opinionated about which particular method should be chosen for power-phase analysis, it demands that a common framework should be decided for each research context so as to make the results easily comparable across studies. Consistency across signal processing methods can alleviate restrictions for federated machine learning methods, e.g., where data may not be shared across international borders, but model parameters may be.

## 5. Conclusions

Methodological differences across EEG processing studies make it difficult to compare results between them. In addition to that, there have been reports on inconsistent results across studies. More often than not, the methodological differences are held accountable for inconsistent results. This study sets out to validate this claim by running a set of methods with multiple selection choices on EEG signals obtained from two datasets. Statistical analyses were performed on a total of 60 subjects (45 from Dataset 1 and 15 from Dataset 2) to find out the effect of the selections. The results indicated that selection of EEG transformation method and time-window may influence the PSD quantities. Equally important, choices of EEG transformation method and filter algorithm may affect phase quantities. Moreover, the combination of selections of multiple methodological choices has the capability to introduce interaction effects, causing results to differ from that with other studies. Therefore, it was proposed that a consistent set of choices should be made in this signal processing domain. A consistent processing pipeline will not only allow researchers to compare different studies, but also offer a way to share model parameters, improving reproducibility for authors.

## Figures and Tables

**Table 1:** The results of 40 linear mixed effect models (20 for Dataset 1 and 20 for Dataset 2), where the response variable was the PSD. The effects of choices in artifact removal, EEG transformations, filters, time windows, and PSD estimation methods are outlined in the table. The effects are shown separately for each frequency bands. Significant *p*-values are marked with asterisks (*).

Factor	Model Number	Band	Dataset 1		Dataset 2	
			*p*	F	*p*	F
Artifact removed	1	Theta	<0.001 *	23.21	0.0071 *	7.24
	2	Mu	0.025 *	5.03	0.2498	1.32
	3	Beta	<0.001 *	17.53	<0.001 *	35.78
	4	Gamma	<0.001 *	1347.72	<0.001 *	48.9
EEG transformation	5	Theta	<0.001 *	5341.06	<0.001 *	580.31
	6	Mu	<0.001 *	2777.1	<0.001 *	1084.61
	7	Beta	<0.001 *	5470.85	<0.001 *	1337.18
	8	Gamma	<0.001 *	6507.23	<0.001 *	915.71
Filter	9	Theta	0.2208	1.5	<0.001 *	73
	10	Mu	0.4626	0.54	<0.001 *	168.87
	11	Beta	<0.001 *	11.43	<0.001 *	169.21
	12	Gamma	<0.001 *	1320.39	<0.001 *	3175.94
Time window	13	Theta	0.3924	0.73	<0.001 *	106.38
	14	Mu	0.5058	0.44	0.002 *	9.53
	15	Beta	<0.001 *	19.87	<0.001 *	195.56
	16	Gamma	<0.001 *	720.45	<0.001 *	1705.33
PSD estimation method	17	Theta	<0.001 *	1546.66	<0.001 *	9500.41
	18	Mu	<0.001 *	1602.82	<0.001 *	3486.92
	19	Beta	<0.001 *	12,867.49	<0.001 *	61,531.19
	20	Gamma	<0.001 *	86,225.6	<0.001 *	161,573.05

**Table 2:** The results of 48 linear mixed effect models (24 for Dataset 1 and 24 for Dataset 2), where the response variable was the phase of the signal. The effects of choices in artifact removal, EEG transformation, and filter are outlined in the table. The effects are shown separately for each frequency bands. Significant *p*-values are marked with asterisks (*).

Category	Factor	Model Number	Band	Dataset 1		Dataset 2	
				*p*	F	*p*	F
Peaks	Artifact removal	1	Theta	0.8540	0.03	0.9835	0
		2	Mu	0.9414	0.01	0.8791	0.02
		3	Beta	0.7915	0.07	0.9774	0
		4	Gamma	0.7467	0.1	0.8507	0.04
	EEG transformation	5	Theta	0.0016 *	6.43	0.7114	0.34
		6	Mu	0.4775	0.74	0.7152	0.34
		7	Beta	0.4565	0.78	0.8154	0.2
		8	Gamma	0.5925	0.52	<0.0001 *	37.2
	Filter	9	Theta	<0.0001 *	64.94	<0.0001 *	199.87
		10	Mu	<0.0001 *	28.92	<0.0001 *	124.03
		11	Beta	<0.0001 *	19.76	<0.0001 *	91.64
		12	Gamma	0.0534	3.74	<0.0001 *	248.94
Troughs	Artifact removal	13	Theta	0.4906	0.48	0.4763	0.51
		14	Mu	0.918	0.01	0.8828	0.02
		15	Beta	0.9619	0	0.9739	0
		16	Gamma	0.3493	0.88	0.8176	0.05
	EEG transformation	17	Theta	<0.0001 *	16.09	0.3556	1.03
		18	Mu	0.3422	1.07	0.7311	0.31
		19	Beta	0.8887	0.12	0.119	2.13
		20	Gamma	0.0096 *	4.65	<0.0001 *	137.21
	Filter	21	Theta	0.0785	3.1	<0.0001 *	162.35
		22	Mu	0.0095 *	6.74	<0.0001 *	129.54
		23	Beta	<0.0001 *	15.2	<0.0001 *	94.82
		24	Gamma	0.1117	2.53	<0.0001 *	161.87
